# Intranodal Ultrasound-Guided Percutaneous Methylene Blue Injection for the Identification of Leakage Point during Laparoscopic Repair of Refractory Chylous Ascites after Laparoscopic Lymphadenectomy for Kidney Cancer

**DOI:** 10.1155/2022/3817554

**Published:** 2022-11-21

**Authors:** Hugo Otaola-Arca, Patricio Vargas, Daniel Hasson, Marcelo Orvieto, Carmen Niño-Taravilla, Hugo Bermúdez

**Affiliations:** ^1^Clínica Alemana, Santiago, Chile; ^2^School of Medicine, Clínica Alemana-Universidad del Desarollo, Santiago, Chile; ^3^Clinica INDISA, Santiago, Chile

## Abstract

Chylous ascites is an uncommon complication after surgery that can result in malnutrition and immunodeficiency. Therefore, surgical interventions are reserved for refractory patients, and the primary success factor for these interventions is locating the point of leakage, which is often tricky. We describe a case of a 56-year-old male with chylous ascites after laparoscopic radical nephrectomy and lumbo-aortic lymphadenectomy for kidney cancer. The patient was initially managed with dietary modifications and drainage placement. Afterward, lymphography with Lipiodol, percutaneous embolization of the leakage point, and total parenteral nutrition were established. Finally, the patient underwent laparoscopic repair after identifying the leakage point by injecting methylene blue through an inguinal node. Complete resolution was achieved, and no complications related to the procedure were recorded. Intranodal methylene blue injection can be an invaluable tool to identify the point of leakage in selected patients to improve the outcomes of surgical repair of refractory chylous ascites.

## 1. Introduction

Chylous ascites (CA) is the accumulation of chyle within the peritoneum secondary to chylous leakage (CL), which can be developed after major abdominal surgery due to an inadvertent iatrogenic injury of cisterna chyle or one of its main tributaries and can be life-threatening as a result of the secondary disorders (electrolyte imbalance, malnutrition, and immunodeficiency).

Nowadays, no standardized treatment guidelines are available, but classifications based on severity have been used to harmonize its management [1]. Dietary modification (high-protein and low-fat diet, medium-chain triglycerides (MCT) supplements) to reduce the intake of long-chain triglycerides (LCT) is accepted as a first-line approach. Absolute fasting with total parenteral nutrition and somatostatin or analogs is often second-line therapy [2]. Paracentesis or drain placement [3], lymphangiography, and embolization are alternatives for nonresponders [4]. Surgical interventions are reserved for refractory patients. However, the intraoperative location of the leakage point is the most significant limiting factor in the success of the surgical repair.

This article aims to present a case of refractory CA in a patient with kidney cancer and to describe a successful laparoscopic repair of CL after a percutaneous injection of methylene blue in an inguinal lymph node.

## 2. Case Presentation

We present a 56-year-old man with hypertension and hypothyroidism under treatment, without known allergies. On general physical examination, only a body mass index of 33.4 Kg/m^2^ and scars of previous laparoscopic cholecystectomy are noted.

**Figure 1:**
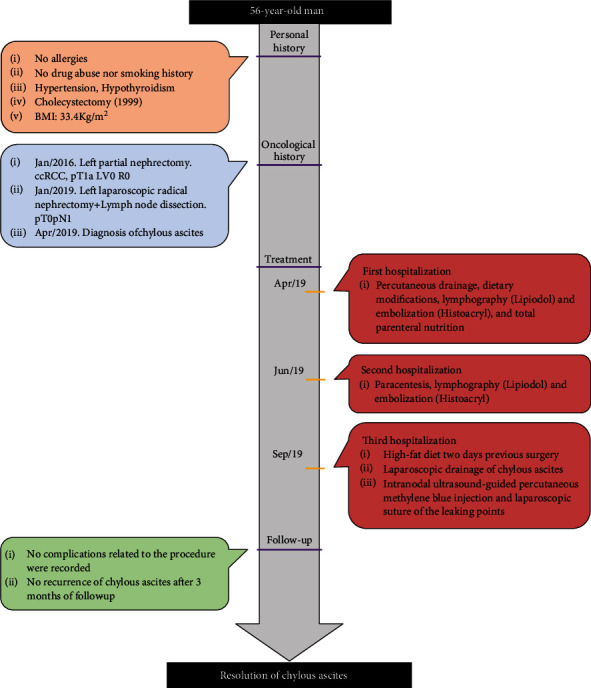
Timeline of interventions and outcomes. BMI = body mass index, ccRCC = clear cell renal cell carcinoma, LV = lymphovascular invasion, R = margin status.

In January 2016, the patient underwent an elective left partial nephrectomy due to an incidental renal tumor (pT1a, clear cell renal cell carcinoma (ccRCC) with negative margins). Unfortunately, the patient presented local lymph node recurrence three years later, and an uneventful left laparoscopic radical nephrectomy (LRN) and lumbo-aortic lymphadenectomy (LALA) were carried out. Metastasis of ccRCC was found in two out of five lymph nodes without evidence of tumor in kidney tissue (pT0pN1).  Figure 1 summarizes the timeline of interventions and outcomes.

**Figure 2:**
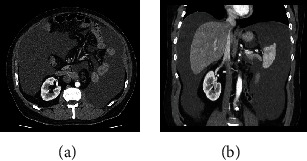
Computed tomography scan. Massive ascites can be seen in the (a) axial and (b) coronal sections.

**Figure 3:**
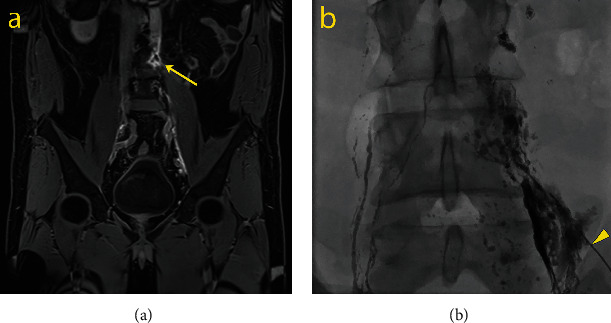
Magnetic resonance lymphangiogram with Lipiodol. Dynamic contrast-enhanced magnetic resonance lymphangiogram (a) with maximum intensity projection and the subtraction of noncontrasted structures shows a leak site at the L4-L5 level (arrow). (b) Shows the percutaneous embolization with Histoacryl® (head arrow) after identifying the leakage point in the lymphography with Lipiodol.

In April 2019, the patient presented to the emergency service complaining of generalized abdominal pain, marked abdominal distension, nausea, and vomiting. Abdominopelvic computed tomography (AP-CT) scan revealed many intra-abdominal free fluids (Figure 2). The patient was diagnosed with CA based on the findings in both diagnostic paracentesis and dynamic contrast-enhanced magnetic resonance lymphangiogram (DCMRL) (Figure 3(a)). Percutaneous drainage was placed (12 L of chyle), and dietary modifications were applied (high-protein and low-fat diet with MCTs supplements). Due to the persistence of an active CL, the point of CL was identified using an intranodal lymphangiography with Lipiodol and embolized with Histoacryl® (Braun) in a 1 : 4 ratio (Figure 3(b)). During follow-up, despite dietary restrictions, the patient presented progressive abdominal distension. Therapeutic paracentesis was performed every 3-4 weeks, evacuating a maximum of four liters per session. A new Lipiodol lymphangiography and uneventful Histoacryl® embolization were performed at the beginning of June. The team underwent surgical treatment because CL remained active despite conservative measures and two embolizations.

**Figure 4:**
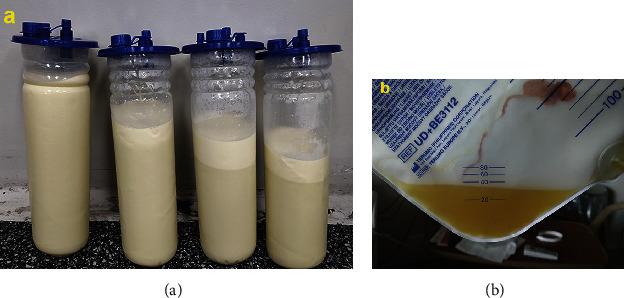
Evolution of the appearance of the abdominal drainage fluid. Milky white ascites obtained during surgery can be seen in the suction receptacles (a). Since the first postoperative day, clear liquid can be seen in the intra-abdominal drainage collecting bag (b).

**Figure 5:**
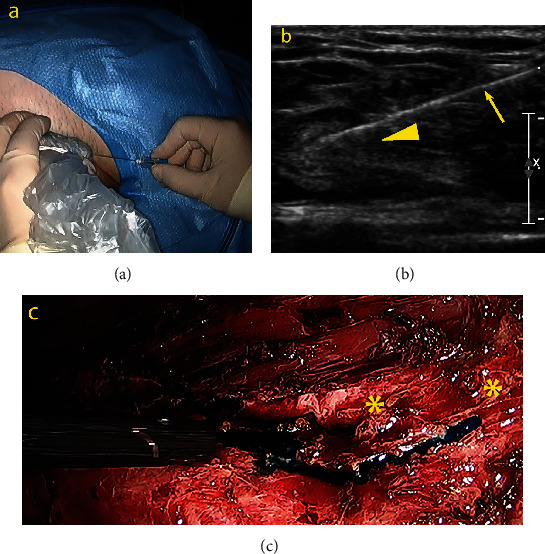
Identification of the point of chylous leakage. External (a) and ultrasound vision (b) of a transnodal injection of methylene blue under image guidance (lymph node, head arrow; needle, and arrow). Two leak points (stars) can be observed in the retroperitoneum (c) after seven minutes of percutaneous administration of methylene blue.

In September 2019, the patient was hospitalized. Two days before the surgery, a high-fat diet was prescribed to increase CA production and identify the point of CL during surgical repair. Laparoscopy was performed in a right lateral decubitus position using two 12 mm and two 5 mm ports inserted in the scars of the previous ports' sites. Intraoperatively, nine liters of chyle was drained (Figure 4(a)). The left Toldt white line was opened, and the left colon was medialized. Despite colon dissection and meticulous observation of the retroperitoneum, the point of CL had not been identified. Subsequently, 20 mL of methylene blue was injected percutaneously in a left inguinal lymph node under ultrasound- (US-) guidance using a standard lymph node US access approach (Figures 5(a)–5(b)). Under sterile conditions, a linear 10-14 MHz probe was used to explore the inguinal region, identifying a lymph node with adequate size and depth for punction. Afterward, a 25G needle was used to access the lymph node under real-time US view. Seven minutes later, two methylene blue leak points into the retroperitoneum were identified in the para-aortic region, 4 cm below the old renal hilum (Figure 5(c)). Endosuturing of both leaking points was done from distal to proximal with a running barbed suture (3/0 Stratafix™, Ethicon) reinforced with titanium clips. A new injection of methylene blue ruled out the persistence of the CL. The procedure is summarized step-by-step in Video S1. First, an abdominal drain was placed (19 Ch Blake). The total operative time was 90 minutes, and the estimated blood loss was 20 mL.

To help the healing of the CL, the patient was under absolute fasting and parenteral nutrition during the first three postoperative days (POD). Additionally, from the third to the sixth PODs, he was fed a protein-rich, low-fat diet and MCT supplements. However, during the immediate postoperative period, the liquid coming from the drainage was clear (Figure 4(b)), and the biochemical analyses confirmed transudative fluid (low triglyceride and protein content). Therefore, the drain was withdrawn on the fourth POD, and the patient was discharged on the seventh POD.

After twelve months of follow-up, our patient had a complete resolution of CA, a good nutritional and performance status, and no immediate or delayed complications related to the procedure were recorded.

## 3. Discussion

We report a case of CA after LRN and LALA for kidney cancer successfully treated with laparoscopic repair after the failure of conservative treatment, where intranodal ultrasound-guided percutaneous methylene blue injection was crucial to identify the leakage point.

CA is an infrequent but significant complication to consider after abdominal surgery. CA may go unnoticed intraoperatively in laparoscopic surgery because the high intra-abdominal pressure created by the pneumoperitoneum can mask leakage from the low-pressure lymph vessels. Various algorithms have been reported [5–9], but no standardized treatment guidelines are available yet; thus, the optimal management of postoperative CA is unclear.

Surgical interventions are reserved for refractory patients. The most common procedure is to suture or clip the point of the CL in the retroperitoneum once it is localized. Direct glue application (e.g., Floseal), argon beam [2], intravenous heparin injection [10], and low-dose radiation therapy [11] have been described as alternatives to suturing. Laparoscopic suture of the point of CL has a success rate of up to 86% [1]. Still, the main pitfall in surgical repair is the failure to identify the point of CL, which is a significant determinant of its success. In this regard, Molina et al., who first reported the laparoscopic approach for CA secondary to lymphadenectomy, could not identify the CL point despite the administration of intravenous indigo carmine and prolonged laparoscopic observation [12]. Thus, the authors performed extensive blinded clipping (50 clips), argon beam coagulation, and fibrin glue (6 units) in the fibrous fatty tissue surrounding the renal hilum.

Some strategies have been described to help localize the point of CL by increasing the production or dyeing the CA [2, 12–16]:

- *Preoperative Strategies*. Ingestion of high-fat diet/milk one or two days before surgery
- *Intraoperative Strategies*. Methylene blue or heavy cream administration through a gastric tube; injection of intravenous indigo carmine during surgery; mesenteric intranodal lymphangiography

In our case, we administered a high-fat diet on the days before surgery to increase chyle production. However, it was impossible to identify CL's point once the retroperitoneum was dissected. Therefore, we administered methylene blue through an inguinal node under ultrasound guidance, and two points of CL could be observed seven minutes later in the para-aortic region. Subsequently, we could perform a laparoscopic suture reinforced with titanium clips selectively on the points of CL. Finally, we administered methylene blue again to the same node, confirming the absence of CL.

Methylene blue is a very versatile drug. As a medication, it helps treat methemoglobinemia, vasoplegic syndrome, ifosfamide-induced encephalopathy, and cyanide poisoning. In addition, it is instrumental in many diagnostic (e.g., bacterial staining and fistula identification) and therapeutic procedures as a dye. However, it should be noted that methylene blue can cause serotonin syndrome in those patients who consume serotonergic psychiatric medications [17], probably because it is a potent reversible monoamine oxidase inhibitor. Therefore, its use should be avoided in these patients, even in small amounts.

It is important to highlight that chylous lymphatic fluid mostly forms after fat absorption in the small bowel and originate from the above part of the body and thus some refractory chylous ascites cannot be detected by lymphangiography from the below. In this scenario, mesenteric intranodal lymphangiography could be useful, as described by Lee et al. [16].

To conclude, identifying the point of the CL is of paramount importance to enable the success of the surgical treatment. In this regard, percutaneous image-guided intranodal methylene blue injection through an inguinal lymph node is an invaluable tool to identify the CL during surgical repair, allowing selective lymphatic ligation.

## Data Availability

Raw data is available upon request from the corresponding author.

## Abbreviations

AP-CT: Abdominopelvic computed tomography
CA: Chylous ascites
ccRCC: Clear cell renal cell carcinoma
CL: Chylous leakage
DCMRL: Dynamic contrast-enhanced magnetic resonance lymphangiogram
LALA: Lumbo-aortic lymphadenectomy
LCT: Long-chain triglycerides
LRN: Laparoscopic radical nephrectomy
MCT: Medium-chain triglycerides
POD: Postoperative day
US: Ultrasound.

## Supplementary Materials

Supplementary Materials Video S1: Identification of the chylous leakage after transnodal methylene blue injection, suturing, and clipping of the leakage.
