# Supplementary material for: Intranodal Ultrasound-Guided Percutaneous Methylene Blue Injection for the Identification of Leakage Point during Laparoscopic Repair of Refractory Chylous Ascites after Laparoscopic Lymphadenectomy for Kidney Cancer
Source: Case Rep Urol. 2022 Nov 21;2022:3817554. doi: 10.1155/2022/3817554 (PMC9705082; doi:10.1155/2022/3817554)
Supplement: Supplementary Materials — Video S1: Identification of the chylous leakage after transnodal methylene blue injection, suturing, and clipping of the leakage. [file 3817554.f1.docx]

You can download the video at the following link.

<https://drive.google.com/file/d/1JzaUsKZr85iE2yCzLNfi4UMZ0L0qfkkE/view?usp=sharing>
